# Amantadin resistant variants of influenza A virus from Flu-like infected suspects in a Children Infectious Research Center in Tehran, Iran

**Published:** 2011-01-10

**Authors:** Zahra Shokati Eshkiki, Fatemeh Fotouhi, Vahideh Mazaheri, Tina Bolurieh, Abbas Jamali, Sedigheh Rafiee Tabatabaee, Masoumeh Tavassoti Kheiri

**Affiliations:** 1 Department of Virology (Influenza Unit), Pasteur Institute of Iran, Tehran 13169-43551, Iran; 2 Children Infectious Research Center, Mofid Hospital, Tehran 15514-15468, Iran

## 

The matrix protein 2 (M_2_) blocker, Amantadin, is approved by FDA to treat and control of influenza A virus infections in children and adults. Recently, some substitutions at amino acid positions are causing the emergence of anti viral drug-resistant strains of influenza virus.

To investigate the frequency of Amantadin resistance among influenza A viruses isolated in children (up to 10 years old) referring to Children Infectious Research Center in Tehran – Iran during the 2008 – 2009 Flu season, 124 samples were collected. Forty cases from them were detected as influenza A virus by Reverse transcriptase polymerase chain reaction (RT-PCR). The Large subunit of hemagglutinin (HA1) and M_2_ genes were amplified by RT-PCR followed by sequencing.

62.5% of positive cases were 0-3 years, 10% of them were 4-6 years, and 27.5% were 7- 10 years. The result shows 70% were male. 50% of positive cases affected sporadic. Clinical symptoms in these positive cases were as follows: Sudden onset 51%, fever 69.4%, headache 21.8%, coryza 82.5%, weakness 54.8%, pharingitis 41.1%, bronchodyspenia 4%, cough 11.3%, agitation 58%, sputum 59.7%, and muscle pain 8%, gastrointestinal problem, 23.4%, otitis 13%. 97.5% of these forty cases did not received influenza vaccine and 40% received antibiotic.

Our data show that Amantadin resistant A/H_3_ N_2_ is caused by a single amino acid mutation that makes a Ser 31 Asp substitution in M_2_ protein. Since these children might not used Amantadin, the drug resistance determined in this project is not related to direct usage of Amantadin by the patient. Continued surveillance is required to elucidate full Amantadin-resistant pattern of influenza virus in Iranian children, especially in the recent pandemics.

